# Clinical and Imaging Phenotypes and Outcomes in a Costa Rican Cohort of Acute Ischemic Stroke Survivors: A Retrospective Study

**DOI:** 10.3390/jcm12031080

**Published:** 2023-01-30

**Authors:** María Lorena Serrano-Castro, Mónica Garro-Zúñiga, Erik Simon, Arturo Tamayo, Timo Siepmann

**Affiliations:** 1 Department of Internal Medicine, Hospital Chacón Paut, Caja Costarricense de Seguro Social, San José 10101, Costa Rica; 2 Division of Health Care Sciences, Dresden International University, 01067 Dresden, Germany; 3 Department of Neurology, Hospital San Juan de Dios, Caja Costarricense del Seguro Social, San José 94088, Costa Rica; 4 Department of Neurology, University Hospital Carl Gustav Carus, Technische Universität Dresden, 01069 Dresden, Germany; 5 Winnipeg Regional Health Authority (WRHA), Section of Neurology, Department of Medicine, The Max Rady Faculty of Health Sciences, Brandon Regional Health Centre, University of Manitoba, Winnipeg, MB R3T 2N2, Canada

**Keywords:** brain, ischemia, AIS, central America, prevention

## Abstract

Background: We characterized clinical and imaging phenotypes and their association with clinical outcomes in acute ischemic stroke (AIS) survivors in the understudied region of Costa Rica. Methods: We conducted a retrospective cohort study in AIS patients treated at a tertiary stroke center in Costa Rica from 2011–2015. Participants underwent detailed phenotyping for cardiovascular risk factors and stroke etiology. We assessed the association of ischemic brain lesion features and clinical outcomes using the Oxfordshire Community Stroke Project (OCSP) classification. Results: We included 684 AIS survivors (60.2% males, aged 68.1 ± 13.6 years, mean ± SD). While the cardiovascular risk profiles and mortality rates of our patients were similar to populations in European and North American countries, only 20.2% of patients with atrial fibrillation (AF) received anticoagulation. On multivariable analysis, patients with total anterior circulation infarct (TACI) displayed an increased risk of complications (OR: 4.2; 95% CI: 2.2–7.8; *p* < 0.001), higher mortality (OR: 6.9; 95% CI: 2.9–16.1; *p* < 0.001) and lower chance of functional independence at discharge (OR: 8.9; 95% CI: 4.1–19; *p* < 0.001) compared to non-TACI. The comorbidity of bronchopneumonia increased the probability of death by 14.5 times. Conclusions: Our observations in a Costa Rican cohort of AIS survivors might help improve local measures for preventing and managing AIS.

## 1. Introduction

Cerebrovascular disease (CVD) is the second leading cause of mortality and one of the main causes of disability worldwide [1,2,3,4]. Remarkably, most of the global stroke burden resides in lower- and middle-income countries [5]. According to the World Health Organization (WHO), nearly 20.5 million strokes occur yearly, of which approximately 5.5 million patients die and 5 million will have some residual permanent disability. The overall incidence of the disease is estimated to be two hundred new cases per 100,000 inhabitants. In addition, this incidence has been increasing for every 10 years above 35 years of age, reaching figures of up to 3000/100,000 inhabitants in people over 85 years of age. The WHO forecasts an increase of approximately 27% in the global incidence of stroke between 2000 and 2025, which is explained by the aging of the population [6,7,8]. The estimations for Western Europe alone are that a new stroke will occur every 7 min and that nearly 1,070,000 new cases will occur per year [9,10,11], whereas in the United States of America, nearly 700,000 new stroke cases are reported yearly, and stroke is the fifth leading cause of all registered deaths [5,12]. The incidence of CVD estimated for Latin America varies between 35 and 183/100,000 inhabitants. However, it is likely that underreporting of cases exists in understudied regions [13,14,15,16]. In 2001, it was estimated that 85.5% of all stroke deaths occurred in developing countries, representing an increasing public health problem. In spite of this, there is a lack of studies characterizing this population. Additionally, some of the few epidemiological stroke studies conducted in Latin America and the Caribbean have reported incidence and prevalence values as well as behavioral patterns that differ from those reported in developed countries [15,16].

In Costa Rica, CVD is the second leading cause of death due to circulatory system diseases, with 1166 deaths from this cause reported during 2013 and 1470 deaths during 2019 [17,18]. In 2002, an epidemiological study on the incidence and risk factors of CVD in Costa Rica at one of the main hospitals in the country showed figures quite different from those reported in the world literature with incidence values for the years 1999 and 2000 of 16.71 and 16.08 per one hundred thousand inhabitants, respectively [19]. Another study published in 2016 analyzed mortality from CVD in Costa Rica in the period from 1920 to 2009 and found that mortality figures showed an upward trend from 1920 to 1969, while mortality rates from 1970 to 2009 decreased for both sexes, a trend that was mostly seen in the early 1990s. The percentage contribution of CVD mortality to the country’s overall mortality also showed a downward trend since 1990. Both crude and adjusted mortality rates found were lower than those reported in other countries in the Latin American region [20]. Better health coverage and CVD risk factor control has been progressively implemented in the country since 1940 with the creation of the social security national health system Caja Costarricense de Seguro Social (CCSS). The further implementation of Rural Community Health Programs since 1973 and 1976, the Family Community Health Programs created since 1987, and the Basic Integral Health Teams established since 1994 as part of an integral health sector reform that reinforced primary health care throughout the country and at the same time strengthened the hospital network, have been probably crucial to determine this reduction in CVD mortality rates observed during the period 1970 to 2009. However, for the period 2009–2019, an upward trend has been observed with incidence values reaching from 70.6 to 88.6 per one hundred thousand inhabitants and mortality rates from 21.4 to 31.2, respectively. This trend can be explained by the aging of the population since the age group of 60 and more years has increased from 7.6% in 2000 to 9.02% in 2009 and 12.8% in 2019, with estimations of an additional increase to 15.7% in 2025 [17,18,20].

The term “stroke” refers to acute brain disease generically encompassing a number of disorders including cerebral ischemia, intracerebral hemorrhage, and subarachnoid hemorrhage. Depending on the nature of the lesion, ischemic stroke is the most frequent type, corresponding to approximately 80% of all strokes [21]. The Oxfordshire classification proposed by Bamford et al. and adopted by the OCSP in 1991, is a universally used clinical classification of ischemic stroke that correlates the clinical manifestations present in the acute phase with the extent and topographic location of the lesion in tomographic findings [22,23,24,25,26,27]. Several studies have also shown that this classification has a good inter-rater reliability [28,29,30]. It categorizes stroke into four subtypes: total anterior circulation infarct (TACI), partial anterior circulation infarct (PACI), lacunar infarct (LACI), and posterior circulation infarct (POCI). This classification has been deemed especially useful for its simplicity, trouble-free application, and predictive forecast value. According to OCSP, the TACI is considered the most severe clinical syndrome being associated with high mortality and significant disability, whereas the LACI is associated with a good outcome, having the best chance of survival despite causing progressive motor deficits and disability in time [31,32,33].

In Costa Rica specifically, there is a lack of preliminary studies describing the sociodemographic, epidemiological, and clinical characteristics of ischemic stroke patients according to the clinical presentation groups proposed by the OCSP classification. A recent study published in 2017 found that the main characteristics of patients with ischemic stroke in Costa Rica do not differ from the world literature, with arterial hypertension being the most frequent risk factor [34]. In 2018, another study on the epidemiology of CVD in Costa Rica found that the baseline characteristics and distribution of cardiovascular risk factors were similar to findings from other studies in Hispanic populations [35].

A better characterization of CVD in Costa Rica might help identify distributions of cardiovascular risk profiles and cerebrovascular phenotypes among stroke survivors in this still understudied region. Adopting preventive and therapeutic measures based on these data might in turn help improve stroke prevention and clinical stroke care. Here, we aimed to characterize the association of ischemic brain lesion features and clinical outcomes, as well as cardiovascular risk profiles in a Costa Rican population of stroke survivors.

## 2. Materials and Methods

### 2.1. Study Design, Setting, and Patients

We performed a retrospective observational cohort study in adult patients with acute ischemic stroke (AIS), according to the OCSP diagnostic categories, who were treated from August 2011 to December 2015 at the San Juan de Dios Hospital (HSJD) Stroke Unit, Costa Rican Social Security Institution (CCSS). The CCSS corresponds to the national public health system in Costa Rica that has universal and almost complete (94%) health coverage. It consists of a network formed by 30 hospitals and more than 1000 primary and secondary health-care centers around the country, including 250 clinics. Only 3 hospitals are considered as third-level hospitals and one of them is the HSJD. This hospital is located in San José, the capital city, which has a population of 1.46 million inhabitants representing 30% of the country’s total population. It offers multidisciplinary medical care with a maximum spectrum of medical services including an emergency room with 24/7 availability of different medical specialists, medical and surgical intensive care units, and a stroke unit. Standard institutional work up for stroke includes Doppler ultrasonography, echocardiography, long-term continuous electrocardiography (ECG), computed tomography (CT) scanning, computed tomography angiography (CTA), and magnetic resonance imaging (MRI) under request. During our study, intravenous (IV) thrombolytic therapy with recombinant tissue plasminogen activator (rtPA) was not available and endovascular reperfusion therapy (EVT) was scarcely performed. 

A hospital-based prospective stroke registry with data from the HSJD Stroke Unit was initiated in 2011 to evaluate patient characteristics and health care data to the evolution of local stroke care longitudinally. Data collection and outcome evaluation were performed by experienced vascular neurologists. 

The database was accessed to retrieve the sociodemographic and clinical information in our study population of AIS survivors by an investigator (MSC). Stroke subtypes according to the OCSP criteria were classified on admission based on the main neurological deficits. We excluded patients with incomplete information on our study variables. Our study was performed in compliance with the Reporting of Studies Conducted using Observational Routinely Collected Data (RECORD) statement. The (REF) checklist can be found in Supplementary Materials.

### 2.2. Ethical Standard

The study was approved by the Scientific Ethics Committee of the San Juan de Dios Hospital, Costa Rican Social Security Institution (CEC-HSJD-CCSS-Session #005-2018) prior to implementation. It was conducted under strict adherence to ethical measures and confidential handling of the information in compliance with the current version of the Declaration of Helsinki. The source of the resulting data corresponded entirely to the HSJD stroke registry of the neurology service of the HSJD, CCSS. All data were anonymized prior to the analysis.

### 2.3. Data Extraction and Variables

Data information was obtained from the patient’s hospital clinical charts and compiled in the HSJD stroke registry using an Excel (Microsoft) spreadsheet. Data collection was conducted by an investigator (MSC).

We extracted sociodemographic data on age, sex, educational level, and employment status. We also retrieved data on OCSP category (TACI, PACI, LACI, POCI), as well as cardiovascular risk factors and comorbidities by reviewing medical notes on self-reported previous medical history. Additional data was extracted on smoking, sedentary lifestyle, early-age vascular family history, hypertension, diabetes mellitus, AF, ischemic heart disease, cardiac valve replacement, dyslipidemia, chronic kidney disease, hypothyroidism, and cancer. Furthermore, we extracted data on previous drug treatment including antihypertensive agents, euglycemic drugs, cholesterol-lowering agents, platelet inhibitors, and anticoagulants. We also reviewed and extracted data on number of previous strokes and number of previous transient cerebral ischemic attacks (TIAs), as well as on type and number of in-hospital complications including bronchopneumonia, assisted mechanical ventilation (MV), cerebral edema, cerebral hemorrhagic transformation, urinary tract infection (UTI), hydro-electrolytic imbalance (HEI), and neurogenic dysphagia with percutaneous endoscopic gastrostomy requirement (PEG), as well as in-hospital mortality and length of hospital stay. Data on neurological deficits quantified via National Institutes of Health Stroke Scale (NIHSS) score at admission and discharge, and functional deficits assessed via modified Rankin Scale (mRS) score at admission and discharge, were also extracted.

### 2.4. Statistical Analysis

We used the STATA^®^ software package (Version 13 StataCorp LP, Texas, TX, USA) for all analyses. The level of statistical significance was defined as 0.05. (A two-tailed *p* < 0.05 indicated statistical significance). Descriptive and inferential statistical evidence was used for data analysis. Statistical test selection was done according to the type of variable. Contrast and homoscedasticity tests were applied (Box plot, Shapiro–Wilk, and Levene) for normality criteria. Quantitative variables with normal distribution were reported as means with 95% confidence intervals or standard deviation. Quantitative variables whose distribution was not normal were reported as medians with values of the 25th and 75th percentiles. Comparisons between quantitative variables with normal distribution were made using Student’s t-test and ANOVA. Non-parametric tests such as Mann–Whitney, Wilcoxon, or Kruskal–Wallis were used for quantitative variables with abnormal distribution. Estimation of absolute and relative frequencies was made for the categorical variables, which were compared using the chi-square test (×2) or the Fischer test. Post hoc multiple comparisons to compare any two OCSP categories for significant differences of the clinical outcomes was performed using ×2 or Fischer´s exact test for categorical variables, whereas a t-test or the corresponding non-parametric test was applied for pairwise comparisons between quantitative variables. Bonferroni correction was performed. Statistical significance was determined as a *p* value <0.008 (0.05/6) for the post hoc multiple comparisons. A multiple linear regression, as well as a multinomial logistic regression, was performed in order to model the probability that a patient would present the event according to their diagnostic category and determine its predictive value. The models were adjusted to control for possible confounding factors or interactions. In accordance with classical predictive models, the selection of covariates in the model was strictly selected. Linear regression analysis was performed to determine variables associated with a longer hospital stay. The covariates selected in the model were non-use of antihypertensives, PACI group, presence of complications, and UTI. The multivariate analysis to determine associated factors independently and significantly with the dependent variables of mortality, complications, stroke severity, and disability was performed using multiple logistic regression techniques. The covariates selected for the adjusted mortality analysis were: age, gender, AF, anticoagulation in AF, presence of complications, a complication of bronchopneumonia, a complication of cerebral edema, a complication of MV, and unemployment condition. The selected covariates for the adjusted analysis of complications were anticoagulation in AF, prior treatment, unemployment status, NIHSS admission, and days admitted.

Since the main differences in the clinical outcomes were detected between TACI and the other groups, these three groups were analyzed as non-TACI groups in the multivariable regression. The median NIHSS score > 10 was established as the demarcation line for severity and the mean mRS = 2 at discharge was determined as the cut-off for good functional outcomes. The interactions of baseline NIHSS score with OCSP subtype on the clinical outcomes were analyzed by multivariable logistic regression modeling. Full data analysis was performed and no data imputation was performed because in most of the studied variables, the lack of data was null or scarce (less than 10%) in relation to the sample size. Those variables with missing data more than 10% had no significant difference in the missing amount of data between the groups. Data loss was completely at random. 

## 3. Results

### 3.1. Demographic Characteristics and Cardiovascular Risk Profiles

From a total of 822 stroke patients collected in the studied period, 77 were excluded due to CT- or MRI-based diagnosis of cerebral hemorrhage. From the remaining 745 AIS cases, 684 cases that had the OCSP diagnostic category were included. The PACI group corresponded to the most frequent diagnostic category (36.40%), followed by the LACI (33.2%), POCI (16.2%), and TACI (14.2%) groups. The male gender prevailed in relation to the female gender in all groups with an average of 60.23%. This trend was predominant in the POCI group in relation to the other groups with an average of 74.8% (*p* < 0.01), while the TACI group presented a lower predominance of the male gender corresponding to 53.6% (*p* < 0.01). No difference in age was found between the groups whose average age range was 68.13 ± 13.63 years. A total of 69.31% of the cases presented an educational level corresponding to the category of primary education or less, without finding a difference between the groups (*p* = 0.098).

The level of unemployment was higher in the TACI group, which had an unemployment rate of 84.7% in relation to the group average rate, which was 74.48% (*p* = 0.039). No difference was found between the groups regarding the type of occupation reported. Systemic arterial hypertension was the most frequently observed risk factor (78.52%) with no significant difference observed between the diagnostic categories. Other prevalent risk factors were sedentary lifestyle (51.4%), smoking (41%), dyslipidemia (40.7%), diabetes mellitus (35.6%), and AF (13.7%).

No differences were detected between the groups in the frequency of other medical pathologies or risk factors except in the case of AF, which was more frequent in the TACI group (30.5%) (*p* < 0.01). (Table 1) Of the total number of patients with AF, only 20.2% were on anticoagulant therapy at the time of the event, with the TACI group having the lowest frequency of anticoagulation in this subgroup (14.3%) (*p* < 0.01). The reported frequency of other treatments prior to the event was similar between groups. Patients representing 29.8% of the cases received aspirin, 5.5% received clopidogrel, 27.7% received a statin, which corresponded to 84.6% of cases diagnosed with dyslipidemia, 15.7% received an antihypertensive, corresponding to 20.4% of the total cases diagnosed with hypertension, and 5.8% received some euglycemic treatment, which corresponded to 16.9% of the total cases diagnosed with diabetes mellitus. However, the number of patients without treatment for all causes at the time of the event was found to be higher in the TACI group (55.6%) (*p* < 0.01). 

No patient underwent intravenous thrombolytic therapy with recombinant tissue plasminogen activator (rtPA) during in-hospital course, as this therapy was not yet available in the hospital at that time. The presence of other medical comorbidities such as all-cause heart disease, cardiac valve replacement, kidney failure, cancer, or hypothyroidism was similar between groups. 

A history of a previous stroke was reported in 18% of cases and TIA in 3.6% of cases, with no finding of difference between groups in the frequency of any of these events (*p* = 0.81 and 0.38 respectively). Results are detailed in Table 1.

### 3.2. Clinical Outcomes

The reported hospital-acquired complications corresponded to 17.4%, with the TACI group presenting complications with the highest frequency (45.4%) and the LACI group with the lowest frequency (7.5%) (*p* < 0.01). The reported complications that prevailed were UTIs (3.5%), bronchopneumonia (3.4%), hemorrhagic transformation (3.4%), neurogenic dysphagia with PEG tube requirement (2.9%), MV (2%), cerebral edema (1.9%), and hydro-electrolyte imbalances (HEI) (1.8%). The TACI group had a higher incidence of bronchopneumonia (12.4%) (*p* < 0.01), MV requirements (7.2%) (*p* < 0.01), PEG tube requirements (14.4%) (*p* < 0.01), UTI (9.3%) (*p* < 0.01), and cerebral edema (7.2%) (*p* < 0.01). No difference was found between groups in the incidence of other medical complications recorded as hemorrhagic transformation (*p*:0.31) and HEI (*p*:0.45). Results are detailed in Table 2.

The overall in-hospital mortality was 5.9%. Patients in the TACI group displayed the highest mortality (24%), whereas patients in the LACI group had the lowest mortality (0.4%) (*p* < 0.01). Results are detailed in Table 3. 

The main mortality causes were bronchopneumonia (17/40: 42.5%), cerebral edema (10/40: 25%), septic shock (3/40: 7.5%), cerebral hemorrhagic transformation (3/40: 7.5%), UTI (2/40: 5%), and CHF (1/40: 2.5%). Although the TACI group showed a higher frequency of bronchopneumonia, cerebral edema, septic shock, urinary tract infection, and cardiac heart failure as causes of death, the correlation analysis did not reach significance due to a limited sample size of this subgroup. 

The number of days of hospitalization was similar between groups (mean: 9.18 ± 0.54 days) but when excluding the deceased cases from the analysis, we found that the PACI group and the TACI group presented a longer hospital stay (10.92 ± 1.52 days and 10.82 ± 1.75, respectively) (*p* = 0.01).

The NIHSS scale analysis at admission to the Emergency Service and at hospital discharge showed that the TACI group displayed more severe neurological deficits both at admission (*p* < 0.01) and at discharge (*p* < 0.01), compared to the other stroke types (PACI, LACI, POCI). They also displayed lower change in neurological deficits from baseline to discharge. (*p* < 0.01). Results are detailed in Table 4.

By joining the PACI, LACI, and POCI groups, considering them as non-TACI groups and comparing them with the TACI group, it was found that the TACI group presented more severe neurological deficits both at hospital admission and discharge (*p* < 0.01). Results are detailed in Table 5.

Patients in the TACI group showed higher disability on the mRS compared to AIS survivors who classified as PACI, LACI, or POCI. Functional outcome prior to admission was similar between groups. Results are detailed in Table 6 and Table 7 both at admission and discharge (*p* < 0.01).

After multivariable adjustment, the TACI group, as compared to the non-TACI group patients, showed an increased risk of complications (OR: 4.179; 95% CI: 2.227–7.841; *p* < 0.001), higher mortality (OR: 6.9; 95% CI: 2.9–16.1; *p* < 0.001), higher stroke severity at admission (OR: 19.11; 95% CI: 10.75–33.95; *p* < 0.001), and poorer functional independence rate at discharge (OR: 8.85; 95% CI: 4.12–19.04 *p* < 0.001). Results are detailed in Table 8.

The presence of bronchopneumonia conferred a probability of death 14.5 times higher among those who had this complication. The TACI group and the NIHSS score were both predictors of the presence of hospital-acquired complications. The adjusted multivariable logistic regression model with interaction showed no interactions of the severity of neurological deficits at baseline with OCSP subtypes on the risk of complications, in-hospital mortality, and the functional independence rate at discharge. Multiple linear regression analysis found no predictive factors for a longer hospital stay. Results are detailed in Table 9.

## 4. Discussion

While the results of our analysis of OCSP-based AIS subtypes in the stroke registry cohort of a tertiary Costa Rican hospital is overall comparable to well-studied Western regions, the absence of routine IVT/EVT treatment, the low rate of anticoagulation in AF patients, and the patterns of cardiovascular risk factors identified may help improve local strategies for preventing and treating stroke in Costa Rica and comparable regions [36,37,38,39,40,41,42].

In this study, we could confirm that the TACI group had an unfavorable outcome as compared with the other groups, which can be explained by the anatomical and physiological characteristics of this type of infarct which usually implicate an extensive area of cerebral ischemia [22,25,32,33]. Another finding was that the mortality rate was higher in the POCI group than in the PACI and LACI groups, which may be due to the presence of important vital centers in the corresponding posterior circulation area. Moreover, in-hospital morbidity and mortality rates were lower in the LACI group compared to the other groups, as expected in view of their small size and non-cortical location in concordance with the described natural history in this stroke subtype [22,38,39]. Despite their small size, LACI patients have been associated in follow-up studies with long term disability due to underlying small vessel disease and accumulation of multiple lacunar infarctions [22,23,24]. Since this study was a short-term follow-up of patients during hospital evolution and probably because the reported previous TIA and stroke rates were low, we were not able to detect this type of outcome in LACI patients, but otherwise, a favorable outcome was observed compared to the other OCSP subtypes. 

Another finding of this study was that the PACI group corresponded to the most frequent diagnostic category (36.30%) followed by the LACI (33.2%) and the POCI groups (16.2%) respectively, whereas the TACI group corresponded to the least frequent (14.2%). These results are similar to reports from other centers [22]. However, some studies have reported a lower frequency of LACI cases, probably because most of the minor strokes were treated in community hospitals instead of tertiary hospitals [34]. In Costa Rica, most of the strokes are eventually referred to tertiary hospitals, which could explain a higher proportion of patients with LACI as compared to some studies.

Although the male gender prevailed in all groups, the proportion of women was higher in the TACI group in relation to the rest of the groups (46.39%). Several studies have shown that stroke mortality is higher among older women [43,44], although no significant association was found between OCSP subtype and age by gender in this study. 

The mean age of stroke was similar between the stroke subtypes and comparable to reports from developed countries. Other stroke studies in Costa Rica have also reported a higher mean age of onset of stroke compared to other developing countries, which can be explained by the available universal public health care system (CCSS) which covers approx. 94% of the total population, and also by the current long life expectancy in the country (80.3 yrs.) [17,34,35]. An earlier onset of stroke and mortality rates particularly in the age group from 45–64 years has been reported to be even four to five times higher in other developing countries in the Americas than in Costa Rica [20]. This may be due to low health care access, higher prevalence, and inadequate control of cardiovascular risk factors in some countries of the region.

The unemployment rate was significantly higher in the TACI group, which is consistent with studies describing an association between lower socioeconomic status, unemployment, and increased stroke incidence, as well as higher morbidity and mortality from stroke [45,46,47,48,49]. The underschooling average rate was very high in all the subtypes, which has also been considered an important sociodemographic risk factor associated with stroke incidence and severity [49,50]. However, no association was found between low educational level and any of the OCSP subtypes. This may be explained due to the high access to primary health care in the country among different social strata. On the other hand, with the study center being a public hospital, the majority of the served population corresponds to low and medium socioeconomic levels. This might explain the high underschooling rate observed in comparison to the average country’s educational level, estimated as 8.7 mean years of schooling with 57.8% of the population over 15 years of age having one or more years of secondary studies [17].

The prevalence of risk factors was similar to the findings of other studies, with hypertension being the most frequently observed risk factor in all the diagnostic categories [51,52,53]. The TACI group was associated with a higher number of patients without treatment for all causes of studied comorbidities related to cardiovascular risk. This finding reinforces the known impact of hypertension in stroke incidence but may also suggest a worse prognosis for those patients without an adequate control of cardiovascular diseases. 

AF was more common in the TACI group, and anticoagulant therapy in this subgroup was less common in the TACI group compared to the other groups. This finding goes in accordance with the high frequency of cardioembolic underlying mechanism in a TACI [33,54,55,56,57]. AF has been described as causing approx. 20% of all ischemic strokes. Additionally, strokes related to AF have a worse clinical prognosis and higher mortality, which is consistent with the greater association of AF observed in the TACI group. Several studies, including the Framingham Study and data from the OCSP, have consistently found this association between AF and stroke with a more severe presentation and higher fatality rate as compared to sinus rhythm [54,58,59,60]. Since anticoagulant therapy is the reference therapy for prophylaxis of cardioembolic ischemic stroke in patients with AF [61], the lower observed frequency of this therapy in the TACI group in relation to the other groups may explain in part this form of presentation. These findings, in conjunction with the low rate of anticoagulation in any stroke subtype, denote the high importance of an adequate management of stroke risk factors, especially hypertension and atrial fibrillation [62,63,64,65].

In this study, the TACI group was strongly and independently associated with a more severe clinical presentation and worse prognosis evidenced by a greater number of hospital complications, longer hospital stay, higher in-hospital mortality, greater disability, and functional impairment associated with the event. According to the OCSP, a TACI is correlated with higher mortality and significant disability in most survivors whereas patients with a LACI had the best chance of survival, a trend that was also observed in this study. We could also observe that mortality was higher in the POCI group than in the PACI and LACI groups, which has also been described mainly due to large-artery atherosclerosis and to the presence of important vital centers in the posterior circulation area [22,23,24,25,31,32,33].

After multivariable adjustment, it was found that the TACI group had 6.9 times more probability of death and 4179 times more probability of developing complications than the other categories. The bronchopneumonia complication was more frequently observed in the TACI group and conferred 14.5 times higher probability of death among those who presented it. The patients in the TACI group also exhibited a higher degree of neurological involvement at admission and discharge as measured by the NIHSS scale compared to the patients in the non-TACI group, as well as higher functional disability rate at discharge as measured by the mRS, even after multivariable adjustment. It was also determined that the TACI group and the NIHSS scale predicted the presence of hospital-acquired complications. However, no significant interaction was found between the baseline NIHSS at admission and the OCSP classification on the risk of complications, mortality rate, or functional independence rate. 

Several studies have confirmed that the OCSP clinical classification can accurately predict infarct area and size, and is useful to categorize AIS patients at bedside. Moreover, the OCSP subtype might predict the possibility of complications and prognosis which can be useful to determine the best approach and medical treatment [22,23,24,25,31,32,33]. Patient stratification, even before any arterial imaging is available, may also be useful for further clinical trials where treatment effectiveness may depend on the underlying arterial pathophysiological pattern and prompt treatment instauration.

We could also confirm that the prognosis among the OCSP subgroups differed significantly and that belonging to the TACI group could predict deterioration. However, as this is an observational study, there may be limitations derived from potential biases in the selection of patients and assigned treatments, as well as in the observed associations between variables. An attempt was made to minimize this by applying a logistic regression model. On the other hand, being a study based on hospital records of a single national hospital in Costa Rica with a specific area of patient affiliation according to geographical location, the generalization (external validity) of the results may be affected.

Since data collection was performed when no approved thrombolytic therapy in the social security health system was available and endovascular treatment was scarcely provided, this could have affected the neurological outcomes and highlights the necessity of establishing availability of IVT/EVT to all eligible AIS patients. 

This study is limited by its retrospective monocentric nature. However, we were able to include a relatively large sample of AIS survivors in an understudied region, thus providing data that might narrow the knowledge gap on stroke care deriving from large geographic variance in epidemiological and clinical research. 

## 5. Conclusions

In this study, we were able to characterize cardiovascular risk factor profiles in a large cohort of AIS population treated at a national tertiary hospital in Costa Rica, confirm the usefulness of the OCSP classification in an understudied region, and identify targets to improve AIS prevention and care. 

## Figures and Tables

**Table 1:** Sociodemographic and clinical characteristics by OCSP subtype.

Variable	PACI	LACI	POCI	TACI	Total	*p*-Value
Total population, N (%)	249 (36.4)	227 (33.2)	111 (16.2)	97 (14.2)	684 (100%)	
Mean age	68.04 ± 13.91	68.95 ± 12.51	65.47 ± 14.20	69.50 ± 14.48	68.13 ± 13.63	0.11
Male gender n (%)	143 (57.43)	134 (59.03)	83 (74.77)	52 (53.61)	412 (60.23)	<0.006
Female gender n (%)	106 (42.57)	93 (40.97)	28 (25.23)	45 (46.39)	272 (39.77)	
Analyzed [N (%)] N	173 (69.5)	177 (78)	88 (79.3)	54 (55.7)	492	
Education (primary school or less) n (%)	123 (71.10)	119 (67.23)	55 (62.50)	44 (81.48)	341 (69.31)	0.098
Analyzed [N (%)] N	225 (90.4)	212 (93.4)	105 (94.6)	85 (87.6)	627	
Employment status n (%)	55 (24.4)	57 (26.89)	35 (33.33)	13 (15.29)	160 (25.52)	0.039
Analyzed [N (%)] N	238 (95.6)	225 (99.1)	108 (97.3)	80 (82.5)	651	
Current smoking n (%)	94 (39.50)	85 (37.78)	57 (52.78)	31 (38.75)	267 (41.01)	0.056
Analyzed [N (%)] N	159 (63.86)	174 (76.65)	84 (75.67)	58 (59.79)	475	
Sedentarism n (%)	80 (50.30)	93 (53.45)	44 (52.38)	27 (46.55)	244 (51.37)	0.810
Analyzed [N (%)] N	245 (98.4)	220 (97)	107 (96.4)	89 (91.8)	661	
Hypertension n (%)	188 (76.73)	185 (84.09)	78 (72.90)	68 (76.40)	519 (78.52)	0.080
Analyzed [N (%)] N	234 (94)	222 (98)	104 (93.7)	89 (91.8)	649	
Diabetes mellitus n (%)	77 (32.91)	92 (41.44)	34 (32.69)	28 (31.46)	231 (35.59)	0.165
Analyzed [N (%)] N	203 (81.5)	176 (77.5)	85 (76.6)	79 (81.4)	543	
Hyperlipidemia n (%)	90 (44.33)	73 (41.48)	32 (37.65)	26 (32.91)	221	0.325
Analyzed [N (%)] N	246 (98.8)	220 (97)	109 (98.2)	95 (98)	670	
Atrial fibrillation (AF) n (%)	39 (15.85)	18 (8.18)	6 (5.50)	29 (30.53)	92 (13.73)	0.000
Analyzed [N (%)] N	221 (88.7)	203 (89.4)	94 (84.7)	86 (88.7)	604	
Previous stroke n (%)	38 (17.19)	38 (18.72)	15 (15.96)	18 (20.93)	109 (18.05)	0.818
Analyzed [N (%)] N	230 (92.4)	204 (89.9)	105 (94.6)	78 (80.4)	617	
TIA history n (%)	5 (2.17)	10 (4.9)	3 (2.9)	4 (5.13)	22 (3.56)	0.38
Analyzed [N (%)] N	38 (97.4)	17 (94.4)	6 (100)	28 (96.6)	89 (96.7)	
Anticoagulation in AF n (%)	7 (18.4)	6 (35.3)	1 (16.7)	4 (14.3)	18 (20.2)	<0.01
Analyzed [N (%)] N	248 (99.6)	226 (99.6)	111 (100)	90 (92.8)	675	
Anticoagulation for all causes n (%)	10 (4.03)	8 (3.54)	2 (19.8)	6 (6.6)	26 (3.85)	0.36
Analyzed [N (%)] N	248 (99.6)	226 (99.6)	111 (100)	90 (92.8)	675 (98.7)	
Antihypertensive n (%)	37 (15)	33 (14.6)	22 (19.8)	14 (15.6)	106 (15.7)	0.630
Analyzed [N (%)] N	248 (99.6)	226 (99.6)	111 (100)	90 (92.8)	675 (98.7)	
AAS n (%)	78 (31.45)	65 (28.7)	35 (31.5)	23 (25.5)	201 (29.8)	0.710
Analyzed [N (%)] N	248 (99.6)	226 (99.6)	111 (100)	90 (92.8)	675 (98.7)	
Clopidogrel n (%)	15 (6)	7 (3)	8 (7.2)	7 (7.2)	37 (5.48)	0.240
Analyzed [N (%)] N	248 (99.6)	226 (99.6)	111 (100)	90 (92.8)	675 (98.7)	
Statins n (%)	65 (26.2)	67 (29.6)	30 (27)	25 (27.8)	187 (27.7)	0.870
Analyzed [N (%)] N	248 (99.6)	226 (99.6)	111 (100)	90 (92.8)	675 (98.7)	
Euglycemics n (%)	14 (5.6)	14 (6.2)	10 (9)	1 (1.1)	39 (5.8)	0.120
Analyzed [N (%)] N	248 (99.6)	226 (99.6)	111 (100)	90 (92.8)	675 (98.7)	
Treatment for all causes n (%)	128 (51.6)	119 (52.7)	56 (50.5)	40 (44.4)	343 (50.8)	<0.01

Legend to Table 1. Table of sociodemographic characteristics and cardiovascular risk profiles of ischemic stroke patients according to the Oxfordshire Classification treated in the Stroke Unit of the Hospital San Juan de Dios (CCSS) during the years 2011–2015. Abbreviations: PACI: partial anterior circulation infarcts, LACI: lacunar infarcts, TACI: total anterior circulation infarcts, POCI: posterior circulation infarcts, AF: atrial fibrillation, TIA: transitory ischemic attack, AAS: acid acetylsalicylic. N: population analyzed total. [N (%)] N: number of cases with available data and percentage from population analyzed total. n (%): number of cases with findings and percentage in relation to the total cases with available data in each group. Numbers in parentheses indicate percentages.

**Table 2:** Complications by OCSP subtype.

Complication	PACI N = 249	LACI N = 227	POCI N = 111	TACI N = 97	Total 684	*p*-Value
Presence of complications n (%)	43 (17.3)	17 (7.5)	15 (13.5)	44 (45.4)	119 (17.4)	<0.01
Bronchopneumonia n (%)	8 (3.2)	0 (0)	3 (2.7)	12 (12.4)	23 (3.4)	<0.01
MV n (%)	4 (1.6)	0 (0)	3 (2.7)	7 (7.2)	14 (2.0)	<0.01
Hemorrhagic transformation n (%)	11 (4.4)	5 (2.2)	2 (1.8)	5 (5.2)	23 (3.4)	0.310
Brain swelling n (%)	1 (0.4)	0 (0)	5 (4.5)	7 (7.2)	13 (1.9)	<0.01
PEG n (%)	3 (1.2)	0 (0)	3 (2.7)	14 (14.4)	20 (2.9)	<0.01
UTI n (%)	9 (3.6)	5 (2.2)	1 (0.9)	9 (9.3)	24 (3.5)	<0.01
HEI n (%)	4 (1.6)	2 (0.9)	3 (2.7)	3 (3)	12 (1.8)	0.450

Legend to Table 2. Table of hospital-acquired complications of ischemic stroke patients according to the Oxfordshire Classification treated in the Stroke Unit of the Hospital San Juan de Dios (CCSS) during the years 2011–2015. Abbreviations: PACI: partial anterior circulation infarcts, LACI: lacunar infarcts, TACI: total anterior circulation infarcts, POCI: posterior circulation infarcts, MV: mechanical ventilation, PEG: percutaneous endoscopic gastrostomy, UTI: urinary tract infection, HEI: hydro-electrolyte imbalance. N: population analyzed total. n (%) total number of cases with findings and percentage in relation to the total population analyzed. Numbers in parentheses indicate percentages.

**Table 3:** In-hospital mortality by OCSP subtype.

Mortality	PACI	LACI	POCI	TACI	Total	*p*-Value
	247	227	111	96	681	
Global mortality N (%)	10 (4)	1 (0.4)	6 (5.4)	23 (24)	40 (5.9)	<0.01
Bronchopneumonia n (%)	4 (23.5)	0 (0)	3 (17.6)	10 (58.8)	17 (42.5)	
Brain swelling n (%)	2 (20)	0 (0)	1 (10)	7 (70)	10 (25)	
Septic shock n (%)	1 (33.3)	0 (0)	0 (0)	2 (66.7)	3 (7.5)	
Hemorrhagic transformation n (%)	1 (33.3)	1 (33.3)	1 (33.3)	0	3 (7.5)	0.337
UTI n (%)	0 (0)	0 (0)	0 (0)	2 (100)	2 (5)	
CHF n (%)	0 (0)	0 (0)	0 (0)	1 (100)	1 (2.5)	
Total n (%)	8 (80)	1 (100)	5 (83.3)	22 (95.6)	36 (90)	
Unknown n (%)	2(20)	0(0)	1(16.7)	1(4.4)	4(10)	

Legend to Table 3. Table of in-hospital mortality of ischemic stroke patients according to the Oxfordshire Classification treated in the Stroke Unit of the Hospital San Juan de Dios (CCSS) during the years 2011–2015. Abbreviations: PACI: partial anterior circulation infarcts, LACI: lacunar infarcts, TACI: total anterior circulation infarcts, POCI: posterior circulation infarcts, UTI: urinary tract infection, CHF: cardiac heart failure. N: population analyzed total per group. N (%) total number of cases with findings and percentage in relation to the total population analyzed per group. n (%): number of cases with findings for specific causes of death in each group and percentage in relation to the total number of cases with findings for the same cause in all groups. Unknown n (%): cases with missing data and percentage in relation to the total cases with findings per group. Numbers in parentheses indicate percentages.

**Table 4:** Length of hospital stay and stroke severity by OCSP subtype.

Category N (%)	PACI	LACI	POCI	TACI	Total	*p*-Value
N (%) LOS mean days (CI 95%)	195 (78.3) 10.92 (9.40–12.44)	179 (78.9) 8.18 (6.94–9.42)	84 (75.7) 8.76 (7.59–9.93)	61 (62.9) 10.82 (9.07–12.57)	519 (76) 9.61 (8.85–10.38)	<0.014
NIHSS median (Q1–Q3)						
N (%) Admission	235 (94.4) 7.0 (4.00–10.00)	207 (91.2) 4.0 (2.00–7.00)	95 (85.6) 3.00 (2.00–7.00)	93 (95.9) 17.00 (12.00–21.00)	630 (92.1) 6.00 (3.00–10.25)	<0.01
N (%) Discharge	193 (77.5) 3.00 (1.00–8.00)	185 (81.5) 2.00 (0.00–3.00)	74 (66.7) 2.00 (0.00–4.00)	58 (59.8) 14.50 (11.00–18.00)	510 (74.6) 3.00 (1.00–7.00)	<0.01
N (%) Difference (*p* value)	185 (74.3) 3.00 (*p* < 0.01)	169 (74.5) 2.00 (*p* < 0.01)	65 (58.6) 1.00 (*p* < 0.01)	57 (58.8) 2.50 (0.1)	476 (69.6)	

Legend to Table 4. Table on length of hospital stay and stroke severity according to NIHSS score of ischemic stroke patients stratified by the Oxfordshire Classification treated in the Stroke Unit of the Hospital San Juan de Dios (CCSS) during the years 2011–2015. Abbreviations: PACI: partial anterior circulation infarcts, LACI: lacunar infarcts, TACI: total anterior circulation infarcts, POCI: posterior circulation infarcts, NIHSS: National Institute of Health Stroke Scale, LOS: length of stay. N (%): Total number of cases available in each group (excluding deceased cases in LOS) and percentage with data in relation to the total cases in each group. NIHSS difference was obtained by subtracting the NIHSS discharge score from the NIHSS admission score.

**Table 5:** Neurological deficits in TACI vs. non-TACI stroke survivors.

Category	PACI, LACI, POCI (Non TACI)	TACI	Total	*p*-Value
NIHSS median (IQR)				
N (%) Admission	537 (91.5) 5.00 (2.00–8.50)	93 (95.9) 17.00 (12.00–21.00)	630 (92.1) 6.00 (3.00–10.25)	<0.01
N (%) Discharge	452 (77) 2.00 (1.00–5.00)	58 (59.8) 14.50 (11.00–18.00)	510 (74.6) 3.00 (1.00–7.00)	<0.01

Legend to Table 5. Table of NIHSS scores at hospital admission and discharge of ischemic stroke patients stratified by the Oxfordshire Classification (non-TACI vs. TACI group) treated in the Stroke Unit of San Juan de Dios Hospital (CCSS) during the years 2011–2015. Abbreviations: PACI: Partial anterior circulation infarcts, LACI: lacunar infarcts, TACI: total anterior circulation infarcts, POCI: posterior circulation infarcts, Non-TACI: PACI + LACI + POCI. NIHSS: National Institute of Health Stroke Scale. N (%): Total number of cases available in each group and percentage with data in relation to the total cases in each group.

**Table 6:** Functional outcome by OCSP subtype.

Parameter	PACI	LACI	POCI	TACI	Total	*p*-Value
N (%) Previous mRS 0 n/N (%)	235/249 (94.4) 197/235 (83.8)	212/227 (93.4) 173/212 (81.6)	105/111 (94.6) 95/105 (90.5)	92/97 (94.8) 72/92 (78.3)	644/684 (94.15) 537/644 (83.4)	0.27
N (%) Discharge mRS 0 n/N (%)	166/249 (66.7) 46/166 (27.7)	160/227 (70.5) 68/160 (42.5)	71/111 (64) 29/71 (40.8)	51/97 (52.58) 4/51 (7.8)	448/684 (65.5) 147/448 (32.8)	<0.01
N (%) Discharge mean mRS (min-max)	166/249 (66.7) 2.04 (0–5)	160/227 (70.5) 1.28 (0–6)	71/111 (64) 1.51 (0–5)	51/97 (52.58) 3.86 (0–6)	448/684 (65.5) 1.89 (0–6)	<0.01
N (%) Discharge mean mRS > 2	166/249 (66.7) 64/166 (38.55)	160/227 (70.5) 34/160 (21.25)	71/111 (64) 20/71 (28.17)	51/97 (52.58) 41/51 (80.39)	448/684 (65.5) 159/448 (35.49)	<0.01

Legend to Table 6. Table of functional outcomes according to mRS score of ischemic stroke patients stratified by the Oxfordshire Classification treated in the Stroke Unit of the San Juan de Dios Hospital (CCSS) during the years 2011–2015. Abbreviations: PACI: partial anterior circulation infarcts, LACI: lacunar infarcts, TACI: total anterior circulation infarcts, POCI: posterior circulation infarcts, mRS: modified Rankin Scale. N (%): number of cases with data available and percentage in relation to the total number of cases per group. n/N (%): number of cases with findings in relation to the total cases with available data in each group expressed as a percentage in parentheses.

**Table 7:** Clinical outcomes by OCSP subtype.

Outcome	PACI 249	LACI 227	POCI 111	TACI 97	Total 684	*p*-Value
N (%) Complications n (%) (a)	249 (100) 43 (17.3)	227 (100) 17 (7.5)	111 (100) 15 (13.5)	97 (100) 44 (45.4)	684 (100) 119 (17.4)	<0.01
N (%) LOS mean (CI 95%) (b)	195 (78.3) 10.92 (9.40–12.44)	179 (78.9) 8.18 (6.94–9.42)	84 (75.7) 8.76 (7.59–9.93)	61 (62.9) 10.82 (9.07–12.57)	519 (76) 9.61 (8.85–10.38)	<0.01
N (%) Median NIHSS Admission (c)	235 (94.4) 7.0 (4.00–10.00)	207 (91.2) 4.0 (2.00–7.00)	95 (85.6) 3.00 (2.00–7.00)	93 (95.9) 17.00 (12.00–21.00)	630 (92.1) 6.00 (3.00–10.25)	<0.01
N (%) Median NIHSS Discharge (d)	193 (77.5) 3.00 (1.00–8.00)	185 (81.5) 2.00 (0.00–3.00)	74 (66.7) 2.00 (0.00–4.00)	58 (59.8) 14.50 (11.00–18.00)	510 (74.6) 3.00 (1.00–7.00)	<0.01
N (%) NIHSS Difference (*p* value)	185 (74.3) 3.00 (*p* < 0.01)	169 (74.5) 2.00 (*p* < 0.01)	65 (58.6) 1.00 (*p* < 0.01)	57 (58.8) 2.50 (0.1)	476 (69.6)	
N (%) Previous mRS 0 n/N (%)	235/249 (94.4) 197/235 (83.8)	212/227 (93.4) 173/212 (81.6)	105/111 (94.6) 95/105 (90.5)	92/97 (94.8) 72/92 (78.3)	644/684 (94.15) 537/644 (83.4)	0.27
N (%) Discharge mRS 0 n/N (%) (e)	166/249 (66.7) 46/166 (27.7)	160/227 (70.5) 68/160 (42.5)	71/111 (64) 29/71 (40.8)	51/97 (52.58) 4/51 (7.8)	448/684 (65.5) 147/448 (32.8)	<0.01
N (%) Discharge mean mRS (min-max) (f)	166/249 (66.7) 2.04 (0–5)	160/227 (70.5) 1.28 (0–6)	71/111 (64) 1.51 (0–5)	51/97 (52.58) 3.86 (0–6)	448 (65.5) 1.89 (0–6)	<0.01
N (%) Discharge mean mRS >2	166/249 (66.7) 64/166 (38.55)	160/227 (70.5) 34/160 (21.25)	71/111 (64) 20/71 (28.17)	51/97 (52.58) 41/51 (80.39)	448/684 (65.5) 159/448 (35.49)	<0.01
N (%) Mortality n (%) (g)	247/249 (99.2) 10 (4)	227/227 (100) 1 (0.4)	111/111 (100) 6 (5.4)	96/97 (99) 23 (24)	681/684 (99.6) 40 (5.9)	<0.01

Legend to Table 7. Table of clinical outcomes stratified by the Oxfordshire classification. Abbreviations: PACI: partial anterior circulation infarcts, LACI: lacunar infarcts, TACI: total anterior circulation infarcts, POCI: posterior circulation infarcts, LOS: length of stay, mRS: modified Rankin Scale. Notes: chi-square, Fischer exact test, or t-test was used for post hoc multiple pairwise comparisons. Bonferroni correction was applied (*p* value 0.05/6: 0.008). N (%): number of cases with data available and percentage in relation to the total number of cases per group. n/N (%): number of cases with findings in relation to the total cases with data available in each group expressed as a percentage in parentheses. (a) *p*-value refers to difference between all pairwise comparisons. (b) *p*-value refers to comparison of PACI with LACI. (c) *p*-value refers to comparisons between POCI with PACI and TACI; LACI with PACI and TACI; PACI with LACI, TACI and POCI; TACI with PACI, LACI and POCI. (d) *p*-value refers to comparisons between LACI with PACI and TACI; POCI with TACI; PACI with TACI and LACI: TACI with PACI, LACI and POCI. (e) *p*-value refers to comparisons between LACI with PACI and TACI; POCI with TACI; PACI with TACI and LACI; TACI with PACI, LACI and POCI. (f) *p*-value refers to comparisons between PACI with LACI and TACI; LACI with TACI and PACI; POCI with TACI; TACI with PACI, LACI and POCI. (g) *p*-value refers to comparisons between PACI with POCI and TACI; LACI with POCI and TACI; POCI with LACI, PACI and TACI; TACI with PACI, LACI and POCI.

**Table 8:** Logistic regression analysis of mortality stratified by the Oxfordshire classification.

Outcome	TACI n/N (%)	Non TACI n/N (%)	Unadjusted Analysis Odds Ratio 95% CI *p*-Value		Adjusted Analysis Odds Ratio 95% CI *p*-Value	
Complications	44/97 = 45.36%	75/587 = 12.78%	5.67 (3.55–9.05)	<0.001	4.179 (2.227–7.841)	<0.001
Mortality	23/96 = 23.95%	17/585 = 2.9%	10.53 (5.4–20.6)	<0.001	6.9 (2.9–16.1)	<0.001
NIHSS (>10)	74/93 = 79.57%	83/537 = 15.46%	21.30 (12.22–37.14)	<0.001	19.11 (10.75–33.95)	<0.001
mRs discharge (>2)	41/51 = 80.39%	118/397 = 29.73%	9.69 (4.70–19.995)	<0.001	8.854 (4.12–19.04)	<0.001

Legend to Table 8. Table of logistic regression analysis of mortality stratified by the Oxfordshire classification. Notes: Adjusted for age, gender, AF, anticoagulation in AF, presence of complications, a complication of bronchopneumonia, a complication of cerebral edema, a complication of MV, unemployment condition. Notes: NIHSS admission, clinical classification: TACI, non-TACI. Non-TACI: PACI + LACI + POCI. Abbreviations: TACI: total anterior circulation infarct, PACI: partial anterior circulation infarct, LACI: lacunar infarct, POCI: posterior circulation infarct, NIHSS: National Institutes of Health Stroke Scale, mRS: modified Rankin Scale. n/N (%): Number of cases with findings in relation to the total cases in each group expressed as a percentage in parentheses.

**Table 9:** Logistic regression analysis for the clinical outcomes and interactions, according to the baseline NIHSS score.

Outcomes	NIHSS ≤10		NIHSS >10		*p*-for Interactions
	Odds Ratio (95% CI)	*p*-Value	Odds Ratio (95% CI)	*p*-Value	
Complications	2.155 (0.688–6.747)	0.178	2.754 (1.420–5.341)	0.002	0.303
Mortality	7.96 (1.532–41.416)	0.040	3.195 (1.303–7.834)	0.009	0.289
mRs disch >2	7.215 (1.821–28.593)	0.001	2.42 (0.904–6.486)	0.074	0.059

Legend to Table 9. Table of logistic regression analysis for the clinical outcomes and interactions, according to the baseline NIHSS score. Notes: Adjusted for age, gender, AF, anticoagulation in AF, presence of complications, a complication of bronchopneumonia, a complication of MV, a complication of cerebral edema, a complication of PEG, unemployment condition, education. Abbreviations: NIHSS: National Institute of Health Stroke Scale, mRS: modified Rankin scale discharge.

## Data Availability

The data presented in this study are available on reasonable request from the corresponding authors.

## Supplementary Materials

The following supporting information can be downloaded at: https://www.mdpi.com/article/10.3390/jcm12031080/s1, A record checklist statement is attached as a supplement [66].
