# Supplementary material for: Clinical and Imaging Phenotypes and Outcomes in a Costa Rican Cohort of Acute Ischemic Stroke Survivors: A Retrospective Study
Source: J Clin Med. 2023 Jan 30;12(3):1080. doi: 10.3390/jcm12031080 (PMC9917829; doi:10.3390/jcm12031080)
Supplement: Supplementary file 1 [file jcm-12-01080-s001.zip › jcm-2114867-supplementary.pdf]

RECORD Statement—checklist of items that should be included in reports of retrospective studies

	Item No	Recommendation	Page & Line #
**Title and abstract**	1	(*a*) Indicate the study's design with a commonly used term in the title or the abstract	1; 1-35
		(*b*) Provide in the abstract an informative and balanced summary of what was done and what was found	1; 20-35
		(c) The type of data used should be specified in the title or abstract. When possible, the name of the databases used should be included. If applicable, the geographic region and time frame within which the study took place should be reported in the title or abstract. If linkage between databases was conducted for the study, this should be clearly stated in the title or abstract.	1; 1-35
**Introduction**			
Background/rationale	2	Explain the scientific background and rationale for the investigation being reported	2,3; 42-112
Objectives	3	State specific objectives, including any prespecified hypotheses	3; 106-112
**Methods**			
Study design	4	Present key elements of study design early in the paper	3,4; 114-143
Setting	5	Describe the setting, locations, and relevant dates, including periods of recruitment, exposure, follow-up, and data collection	3,4; 114-173
Participants	6	RECORD 6.1: The methods of study population selection (such as codes or algorithms used to identify subjects) should be listed in detail. If this is not possible, an explanation should be provided.	3,4; 114-143
		RECORD 6.2: Any validation studies of the codes or algorithms used to select the population should be referenced. If validation was conducted for this study and not published elsewhere, detailed methods and results of how study participants were identified from medical records should be provided.	4; 152-173
		RECORD 6.3: If the study involved linkage of databases, consider use of a flow diagram or other graphical display to demonstrate the data linkage process, including the number of individuals with linked data at each stage.	
Variables	7	A complete list of codes and algorithms used to classify exposures, outcomes, confounders, and effect modifiers should be provided. If these cannot be reported, an explanation should be provided.	5; 193-215
Data sources/ measurement	8*	For each variable of interest, give sources of data and details of methods of assessment (measurement). Describe comparability of assessment methods if there is more than one group	4,5; 153-219
Bias	9	Describe any efforts to address potential sources of bias	5; 193 -208
Study size	10	Explain how the study size was arrived at	5; 222-224
Quantitative variables	11	Explain how quantitative variables were handled in the analyses. If applicable, describe which groupings were chosen and why	4,5; 180-186
Statistical methods	12	(*a*) Describe all statistical methods, including those used to control for confounding	4,5; 175-219
		(*b*) Describe any methods used to examine subgroups and interactions	5; 213-215
		(*c*) Explain how missing data were addressed	5; 215-219
		(*d*) *Case-control study*—If applicable, explain how matching of cases and controls was addressed<br>*Cross-sectional study*—If applicable, describe analytical methods taking account of sampling strategy	
		(*e*) Describe any sensitivity analyses	

Data access and cleaning methods	12.1	Authors should describe the extent to which the investigators had access to the database population used to create the study population. RECORD	4; 153-173
	12.2	Authors should provide information on the data cleaning methods used in the study.	
Linkage	12.3	State whether the study included person- level, institutional-level, or other data linkage across two or more databases. The methods of linkage and methods of linkage quality evaluation should be provided.	
Results			
Participants	13*	Describe in detail the selection of the persons included in the study (i.e., study population selection), including filtering based on data quality, data availability, and linkage. The selection of included persons can be described in the text and/or by means of the study flow diagram.	5; 222-224
Descriptive data	14*	(a) Give characteristics of study participants (eg demographic, clinical, social) and information on exposures and potential confounders	5,6; 220-270
		(b) Indicate number of participants with missing data for each variable of interest	6,7; 263-270
		(c) *Cohort study*—Summarise follow-up time (eg, average and total amount)	3; 116 -119
Outcome data	15*	*Cohort study*—Report numbers of outcome events or summary measures over time	7-13 ; 271 - 421
		Case-control study—Report numbers in each exposure category, or summary measures of exposure	
		Cross-sectional study—Report numbers of outcome events or summary measures	
Main results	16	(*a*) Give unadjusted estimates and, if applicable, confounder-adjusted estimates and their precision (eg, 95% confidence interval). Make clear which confounders were adjusted for and why they were included	7-13; 271-421
		(*b*) Report category boundaries when continuous variables were categorized	5; 211-213
		(*c*) If relevant, consider translating estimates of relative risk into absolute risk for a meaningful time period	
Other analyses	17	Report other analyses done—eg analyses of subgroups and interactions, and sensitivity analyses	5; 213-215
**Discussion**			
Key results	18	Summarise key results with reference to study objectives	13-16; 423- 559
Limitations	19	Discuss the implications of using data that were not created or collected to answer the specific research question(s). Include discussion of misclassification bias, unmeasured confounding, missing data, and changing eligibility over time, as they pertain to the study being reported.	15;544-559
Interpretation	20	Give a cautious overall interpretation of results considering objectives, limitations, multiplicity of analyses, results from similar studies, and other relevant evidence	13-16; 423-559
Generalizability	21	Discuss the generalisability (external validity) of the study results	15; 548-551,556-557
**Other information**			
Funding	22	Give the source of funding and the role of the funders for the present study and, if applicable, for the original study on which the present article is based	16; 575
Accessibility of protocol, raw data, and programming code	23	Authors should provide information on how to access any supplemental information such as the study protocol, raw data, or programming code.	16;566, 583-585

Benchimol EI, Smeeth L, Guttmann A et al, RECORD Working Committee. **The REporting of studies Conducted using Observational Routinely-collected health Data (RECORD) statement.** PLoS Med. 2015 Oct 6;12(10):e1001885.
